# Guillain-Barré Syndrome Following Zika Virus Infection Is Associated With a Diverse Spectrum of Peripheral Nerve Reactive Antibodies

**DOI:** 10.1212/NXI.0000000000200047

**Published:** 2022-11-21

**Authors:** Alexander J. Davies, Cinta Lleixà, Ana M. Siles, Dawn S. Gourlay, Georgina Berridge, Wanwisa Dejnirattisai, Carolina Ramírez-Santana, Juan-Manuel Anaya, Andrew K. Falconar, Claudia M. Romero-Vivas, Lyda Osorio, Beatriz Parra, Gavin R. Screaton, Juthathip Mongkolsapaya, Roman Fischer, Carlos A. Pardo, Susan K. Halstead, Hugh J. Willison, Luis Querol, Simon Rinaldi

**Affiliations:** From the Nuffield Department of Clinical Neurosciences (A.J.D., S.R.), University of Oxford, John Radcliffe Hospital, UK; Neuromuscular Diseases Unit (C.L., A.M.S., L.Q.), Neurology Department, Hospital de la Santa Creu i Sant Pau, Universitat Autònoma de Barcelona, Spain; Centro para la Investigación Biomédica en red en Enfermedades Raras–(CIBERER) Madrid (C.L., A.M.S., L.Q.), Spain; Institute of Infection (D.S.G., S.K.H., H.J.W.), Immunity & Inflammation, University of Glasgow, University Place, UK; Target Discovery Institute (G.B., R.F.), NDM Research Building, University of Oxford, Old Road Campus, UK; Wellcome Centre for Human Genetics (W.D., G.R.S., J.M.), Nuffield Department of Medicine, University of Oxford, UK; Center for Autoimmune Diseases Research (CREA) (C.R.-S., J.-M.A.), Universidad del Rosario, Bogotá, Colombia; Departamento de Medicina (A.K.F., C.M.R.-V.), Universidad del Norte, Barranquilla, Colombia; Grupo de Epidemiología y Salud Poblacional (GESP) (L.O.,), School of Public Health, Universidad del Valle, Cali, Colombia; Department of Microbiology (B.P.), School of Basic Sciences, Universidad del Valle, Cali, Colombia; Dengue Hemorrhagic Fever Research Unit (J.M.), Office for Research and Development, Siriraj Hospital, Faculty of Medicine, Mahidol Univeristy, Bangkok, Thailand; Department of Neurology (C.A.P.), Johns Hopkins University School of Medicine, Baltimore, MD; and LifeFactors (J.-M.A.), Rionegro, Colombia; Division of Emerging Infectious Disease (W.D.), Research Department, Faculty of Medicine Siriraj Hospital, Mahidol University, Bangkok, Thailand.

## Abstract

**Background and Objectives:**

Recent outbreaks of Zika virus (ZIKV) in South and Central America have highlighted significant neurologic side effects. Concurrence with the inflammatory neuropathy Guillain-Barré syndrome (GBS) is observed in 1:4,000 ZIKV cases. Whether the neurologic symptoms of ZIKV infection are immune mediated is unclear. We used rodent and human live cellular models to screen for anti-peripheral nerve reactive IgG and IgM autoantibodies in the sera of patients with ZIKV with and without GBS.

**Methods:**

In this study, 52 patients with ZIKV-GBS were compared with 134 ZIKV-infected patients without GBS and 91 non-ZIKV controls. Positive sera were taken forward for target identification by immunoprecipitation and mass spectrometry, and candidate antigens were validated by ELISA and cell-based assays. Autoantibody reactions against glycolipid antigens were also screened on an array.

**Results:**

Overall, IgG antibody reactivities to rat Schwann cells (SCs) (6.5%) and myelinated cocultures (9.6%) were significantly higher, albeit infrequent, in the ZIKV-GBS group compared with all controls. IgM antibody immunoreactivity to dorsal root ganglia neurones (32.3%) and SCs (19.4%) was more frequently observed in the ZIKV-GBS group compared with other controls, whereas IgM reactivity to cocultures was as common in ZIKV and non-ZIKV sera. Strong axonal-binding ZIKV-GBS serum IgG antibodies from 1 patient were confirmed to react with neurofascin 155 and 186. Serum from a ZIKV-infected patient without GBS displayed strong myelin-binding and putative antilipid antigen reaction characteristics. There was, however, no significant association of ZIKV-GBS with any known antiglycolipid antibodies.

**Discussion:**

Autoantibody responses in ZIKV-GBS target heterogeneous peripheral nerve antigens suggesting heterogeneity of the humoral immune response despite a common prodromal infection.

Zika virus (ZIKV) is a member of the *Flavivirus* family that is transmitted by *Aedes* spp. mosquito vector species and in humans typically results in asymptomatic or mildly symptomatic infections. An outbreak of ZIKV infections in French Polynesia in 2013, however, was followed by a spike in the diagnoses of the disabling, acute inflammatory neuropathy Guillain-Barré syndrome (GBS). Early case-control studies established 1 excess GBS case for every 4,000 people infected with ZIKV.^1^ By 2015, the ZIKV pandemic reached South America, where in Colombia, the GBS incidence rates were increased by 210%–350% compared with the pre-ZIKV period.^2,3^ The overall risk estimates range from 1 to 8 GBS cases per 10,000 ZIKV infections.^4^ Recent studies estimate that the total number of ZIKV infections during the 2015–2016 outbreak was much higher than originally reported, suggesting that previous incidence rates of GBS per infection may have been substantially overestimated.^5^

The onset of GBS post-ZIKV infection (ZIKV-GBS) occurred within a median of 6 days after a transient febrile illness, although the peak incidence of GBS cases was observed 3 weeks after the peak of ZIKV diagnoses.^1,2^ Whether this represents an infectious, parainfectious, or postinfectious etiology has been debated. Electrophysiologic studies performed on 37 patients with ZIKV-GBS identified in French Polynesia showed characteristics consistent with the acute motor axonal form of GBS (AMAN).^1^ However, follow-up studies at 4 months were more suggestive of distal demyelination.^6^ In a Colombian series, 70% of ZIKV-GBS cases were classified as acute inflammatory demyelinating polyradiculoneuropathy, suggestive of immune-mediated injury.^7^ ZIKV has also been linked to a number of central and peripheral neurologic complications, all of which may have an immune-mediated etiology.^8^

A potential role for ZIKV-induced immunity is supported by similarities between the ZIKV E protein and human complement component C1q in silico and the presence of anti-C1q antibodies in the sera of animals infected with the virus.^9^ Higher-titer antibody responses to ZIKV have been reported in patients who developed GBS compared with those with uncomplicated infections.^10^ More recently, a higher incidence of antiganglioside antibodies was identified in a Brazilian cohort of patients with ZIKV-GBS compared with uncomplicated ZIKV controls,^11^ suggesting an association between nerve reactive autoantibodies and the peripheral neurologic complications of ZIKV.

The peripheral neurology associated with ZIKV infection may also occur via direct viral toxicity.^12^ ZIKV directly infected peripheral neurons and induced cell death in both mouse and cell culture models,^13^ as well as infecting sensory neurons and Schwann cells (SCs) in dorsal root ganglia (DRG) explants from interferon receptor 1 knockout mice leading to myelin fragmentation and later axonal degeneration.^14^ However, the presence of mononuclear inflammatory cells in a biopsy of the fascicular sural nerve in a male patient with ZIKV-GBS, consistent with classic GBS, and the lack of viral transcript detection by PCR suggest that neurotropism is unlikely to be a major causative factor in the demyelination and axonal degeneration observed within the nerve.^15^

The antibody response is thought to be critical for viral control in ZIKV infection.^16^ We therefore sought to address the potential for humoral immunity in ZIKV-GBS. In this study, we screened sera from a large cohort of patients with ZIKV-GBS and healthy and disease controls for nerve cell reactive immunoglobulins. We used a range of animal and humanized nerve cell cultures we have previously shown capable of detecting nerve cell reactive antibodies in a range of autoimmune diseases^17-20^ and proteomic-based antigen detection methods. Our results show that peripheral nerve reactive autoantibodies are indeed a feature of a subset of patients who developed GBS following ZIKV infection, targeting a broad range of peripheral nerve–related antigens.

## Methods

Full details of this section in eMethods, as well as additional data in eFigures and eTables, links.lww.com/NXI/A760, are available in the Supplement. Any additional methodologic details or data not shown are available on reasonable request to the corresponding authors.

### Patient Cohorts and Sample Acquisition

**Figure 1:**
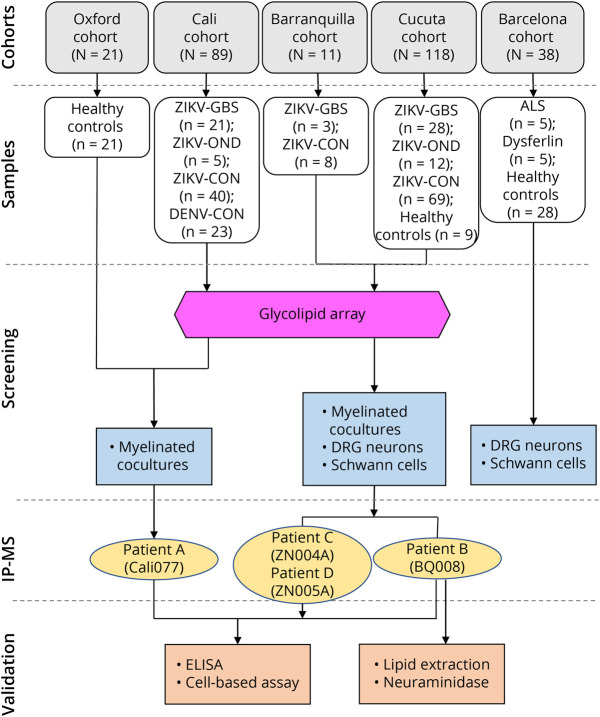
Diagram Illustrating the Patient Cohorts and the Experimental Workflow With Their Serum Samples The numbers of patients' serum samples collected in the different categories of ZIKV-GBS (patients with ZIKV who developed GBS), ZIKV-OND (patients with ZIKV who displayed other neurologic diseases), ZIKV-CON (patients with ZIKV who never developed clinical neurologic disease), DENV-CON (patients with DENV who never developed clinical neurologic disease), ALS (patients with amyotrophic lateral sclerosis, a progressive nervous system disease that affects nerve cells in the brain and spinal cord, causing loss of muscle control), dysferlin (patients with dysferlinopathy, a muscle disease that has a slow progression of muscle weakness and atrophy), and healthy controls (n, number of patient samples). The patients' serum samples were initially screened for their autoantibody reactions against different neuronal cell culture types and from which the sera from patient A to D's serum samples were then tested for their reactions in additional assays.

Serum samples were collected from patients with ZIKV infection both with and without neurologic complications and from other infectious, healthy, and convalescent controls from the Cucuta, Cali, and Barranquilla regions of Colombia, the United Kingdom, and Spain (Figure 1 and eTable 1, links.lww.com/NXI/A760). Patients with ZIKV-GBS were classified according to previously published electrodiagnostic criteria,^7^ with full characterizations previously reported for Cucuta^21^ and Cali cohorts.^3^ Serum samples were collected after treatment, which was given in approximately 60% of patients with ZIKV-GBS (typically infusions of IV immunoglobulin).^3,21^ Samples were aliquoted and stored at −80°C. This study was conducted and reported in accordance with the STROBE guidelines.^22^

### Standard Protocol Approvals, Registrations, and Patient Consents

Sample collection was performed in compliance with Act 008430/1993 of the Ministry of Health of the Republic of Colombia and the institutional review boards of the Universidad del Rosario and Universidad del Valle in Colombia. Additional samples from healthy participants, those with non–immune-mediated neurologic disorders, and nodal/paranodal antibody-positive patients were obtained for use from local collections in Oxford, United Kingdom (South Central–Oxford A Research Ethics Committee approval number 14/SC/0280) or Barcelona, Spain (The Sant Pau Biomedical Research Institute ethical approval number IIBSP-AUT-2016-69). All patients gave written informed consent to participate in the study.

### Rat DRG Neuron and SC Culture and Nerve Section Immunocytochemistry

Rat DRG neurons and SCs were extracted in accordance with Schedule 1 of the UK Home Office Animals (Scientific Procedures) Act 1986 and Animal Ethics' Committee of Hospital de la Santa Creu i Sant Pau. Cells were cultured, and immunocytochemistry was performed as previously described.^23^ Fluorescence signal intensities were scored on a 0–3 scale by 2 independent researchers. Immunohistochemistry on macaque peripheral nerve tissue slides (Inova Diagnostics, Inc., San Diego, CA) was performed and analyzed as previously described.^20^

### Myelinating Cocultures and Immunofluorescence Labeling

Myelinating cocultures were prepared using human induced pluripotent stem cell (hiPSC)-derived sensory neurons and neonatal rat SCs as previously described.^17,24^ Sera from ZIKV-exposed patients were diluted to 1:100 and added to live cocultures for 1 hour at 37°C. Fixed cultures were probed with anti-human IgG, anti-human IgM, or anti-human complement C3c-specific antibodies. Confocal images were assessed for IgG or IgM antibody reactivities by a blinded observer.

### Assessment of Serum-Induced Demyelination

Myelinating cocultures were incubated for 1 hour in complete neurobasal media supplemented with fluoromyelin red. Serum-free myelination medium was then supplemented with or without human anti-ZIKV patients' sera at a 1:100 dilution together with 20% normal human serum (NHS) as a source of complement and added to the cocultures. For the assessment of demyelination in postfixed cultures, a preset of 5 × 5 grid of positions (or 7 × 7 for sparsely myelinated coverslips) at ×20 magnification (0.5 digital zoom) was used for automated acquisition across each coverslip.

### Glycoarray

Serum samples were also screened on a glycolipid microarray as previously described.^25^ For this study, sera were screened against a panel of 16 single glycolipids (GM1, GM2, phosphatidylserine, GM4, GA1, GD1a, GD1b, GT1a, GT1b, GQ1b, GD3, SGPG, LM1, cholesterol, GalC, and sulfatide) and 120 heteromeric 1:1 (vol/vol) complexes, printed in duplicate.

### Immunoprecipitation and Mass Spectrometry

Human sera showing moderate or strong reactivity against rat DRG neurons or myelinating cocultures were used for immunoprecipitation (IP) experiments using the same target cell type, as previously reported.^23^ hiPSC lines from 4 donors were used to generate myelinating cocultures to give 4 biological replicates per serum sample. IP eluates from myelinating cocultures were prepared by chloroform:methanol precipitation and in-solution trypsin digestion. The resultant peptides were then subjected to high-resolution C18 reversed-phase column chromatography and analyzed by data-dependant MS/MS on a ThermoFisher Fusion Lumos mass spectrometer. Significant data points were determined with a permutation-based false discovery rate calculation (threshold = 0.1). Raw data are available via ProteomeXchange with identifier PXD028476.

### Protein Electrophoresis and Western Blot

The lysates from myelinating cocultures, hiPSC-derived sensory neuron monocultures, and primary rat SCs were subjected to electrophoresis in acrylamide gels using MOPS running buffer under denaturing, nonreducing conditions and then transferred to a nitrocellulose membrane. These blots were probed with using patients' sera at a 1:2,500 dilution followed by HRP-conjugated Fc-specific anti-human IgG secondary antibody. The bands detected by Western blotting were aligned in a parallel gel stained with Pierce Imperial Protein Stain and excised for mass spectrometry (MS).

### ELISA

Recombinant human nidogen-1, laminins 111, 121, 211, 221, 411, 421, 511, and 521, prosaposin, and vinculin were coated at 1 μg/mL (100 μL per well) in Maxisorp ELISA plates overnight at 4°C. The ganglioside GM3 ELISA was performed as previously described.^26^ For the detection of anti-ZIKV antibodies, Maxisorp plates were coated with the anti-flavivirus mouse monoclonal antibody 4G2 and performed as previously described.^27^ Bound human IgG antibodies were detected through sequential reaction steps using Fc-specific HRP-conjugated secondary antibodies and substrate. Total human serum IgG levels were interpolated from standard curves according to the manufacturer's instructions (Invitrogen, Cat. 88-50550-86).

### Transfected Cell-Based Assays

HEK293 cells were transfected overnight using JetPEI with mammalian expression vectors encoding human AHNAK2, ANXA2, CD44S, CNTN1, CASPR1, GFRA1, ITGA6, ITGA7, MAG, NEP, NFASC, NKCC1, PRX, and TGBR3 or using Lipofectamine 2000 for human ALCAM, AXL, DPYSL2, GAS6, L1CAM, NCAM1, and NrCAM. Sera were diluted 1:100, and IgG antibody binding was assessed using fluorescent-conjugated anti-human IgG secondary antibodies.

### Statistical Analysis

The results were analyzed in Prism v9.1.0 (GraphPad). Statistical comparisons of the proportions of seropositive patients among the ZIKV groups were performed using contingency analyses with the application of a 2-tailed Fisher exact test for individual group-group comparisons and the χ^2^ test for comparing antibody prevalence between all groups. Analysis of confocal microscopy images was performed by the Student *t* test for comparison of 2 groups and ANOVA for comparison of 3 or more groups, with correction for multiple comparisons where indicated.

### Data Availability

MS proteomics data were deposited to the ProteomeXchange Consortium via the PRIDE partner repository with the data set identifier PXD028476. A summary of MS data and all other anonymized data is available on reasonable request to the corresponding authors.

## Results

### Patient Cohorts

Serum samples were obtained from a total of 218 Colombian participants: 52 patients with GBS and confirmed ZIKV infection (ZIKV-GBS), 17 patients with ZIKV infection and other inflammatory neurologic diseases (ZIKV-OND), 117 ZIKV-infected patients without neurologic complications (ZIKV-CON), and a control group (CON) formed by 23 patients with dengue virus (DENV) infections (DENV-CON) from Cali and 9 healthy controls from Cucuta. An additional 38 serum samples (5 patients with amyotrophic lateral sclerosis, 5 dysferlin patients, and 28 healthy controls) from Spanish participants and 21 healthy controls from the United Kingdom were also used (Figure 1).

Overall, the patients with ZIKV-GBS were slightly older and less often female (eTable 1, links.lww.com/NXI/A760) and showed a modest but significantly higher total serum IgG level (median 4.162 mg/mL; 95% CI 3.473–4.651) than ZIKV-CON (median 3.221 mg/mL; 95% CI 3.004–3.615) and DENV-CON (median 3.210 mg/mL; 95% CI 2.242–3.703) and healthy controls (median 3.051 mg/mL; 95% CI 2.086–3.529), but not ZIKV-OND samples (median 3.601 mg/mL, 95% CI 2.821–3.927) (1-way ANOVA, F(4, 222) = 4.481, *p* = 0.0017) (eFigure 1, links.lww.com/NXI/A760). ZIKV capture ELISA showed the presence of anti-ZIKV IgG in all ZIKV-GBS, ZIKV-OND, and ZIKV-CON patients' sera tested, as well as the DENV patients' sera, likely due to the cross-reactivity of DENV and ZIKV patients' antibodies^27^ (eFigure 1, links.lww.com/NXI/A760).

### Microarray Screening for Glycolipid Complex Antibodies

ZIKV-associated patients' sera were screened against a panel of glycolipids using a combinatorial glycoarray for the presence of IgG (eFigure 2A, links.lww.com/NXI/A760) and IgM (eFigure 2B, links.lww.com/NXI/A760) anti-glycolipid antibodies. Overall, the glycolipid array revealed only weak antibody binding intensities against the panel of 136 unique glycolipid targets, including known gangliosides implicated in GBS, none of which were found to be statistically associated with post-ZIKV GBS. Two ZIKV-GBS samples were found to contain IgG antibodies against various gangliosides at elevated levels, as typically seen in GBS and its clinical variants. This included a single ZIKV-GBS sample with anti-disialosyl IgG (GQ1b, GT1a, and GD3) antibodies normally associated with the GBS subtype Miller-Fisher syndrome and another with anti-GM1 IgG antibodies, typically present in the AMAN subtype.

### Screening Against Rat DRG Neurons and SCs

IgM autoantibodies that were moderate or strongly reactive to rat DRG were observed in proportionally more sera (32.3%: *p* ≤ 0.0001) of patients with ZIKV-GBS compared with patients with ZIKV-OND (16.7%), patients with ZIKV-CON (3.9%), and those from other control (CON) groups (0.0%) (Table 1). On the other hand, moderate or strong IgG autoantibody reactivities against DRG neurons were not statistically different (*p* = 0.1193) between these groups. With the SCs, a greater proportion of moderate or strong IgG (*p* = 0.0348) and IgM (*p* = 0.0121) antibody reactivities were observed in the sera of patients with ZIKV-GBS compared with other patients with uncomplicated ZIKV and control groups (Table 1).

**Table 1:**
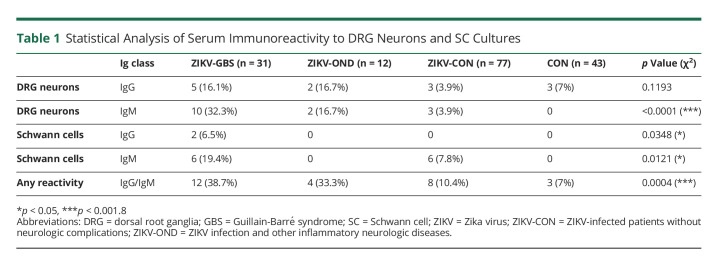
Statistical Analysis of Serum Immunoreactivity to DRG Neurons and SC Cultures

Overall, there was a greater chance of observing any anti-peripheral nerve cell IgG or IgM antibody reactivity in the sera of patients with ZIKV-GBS (38.7%) and ZIKV-OND (33.3%), compared with noncomplicated ZIKV (ZIKV-CON) (10.4%) and non-ZIKV control (CON) (7.0%) groups (*p* = 0.0004, χ^2^ test). In addition, we observed that IgG and IgM antibody binding to DRG neurons or SCs was significantly more common in the sera of patients with ZIKV-GBS than in the sera of the ZIKV-CON group (*p* = 0.0042, Fisher exact test), whereas there was no significant difference in the proportions of grouped anti-ZIKV sera and non-ZIKV sera (*p* > 0.05, Fisher exact test) (see eFigure 3, links.lww.com/NXI/A760, for multiple comparisons).

### Screening Against Myelinating Cocultures

IgM antibody binding to myelinating cocultures was frequently detected with both the sera of patients with ZIKV-GBS and the control group (Table 2 and eFigure 1, links.lww.com/NXI/A760). IgM antibody reactivity was observed against the abaxonal membranes of myelinating SCs, axons, the processes of the nonmyelinating SCs, and outpouchings of myelin (eFigure 4A and 4B, links.lww.com/NXI/A760). Overall, however, there was no significant difference in the proportions of the sera of patients with ZIKV-GBS and controls in terms of the frequency or pattern of positive IgM antibody reactivity (*p* = 0.3374, Fisher exact test) (see Table 2 for multiple comparisons). IgM antibody labeling of myelin outpouchings varied in intensity between the samples (eFigure 4C and 4D, links.lww.com/NXI/A760); however, immunofluorescence intensity at the myelin outpouchings normalized to the internode (eFigure 4E and 4F, links.lww.com/NXI/A760) was again not significantly different between the serum samples of patients with ZIKV-GBS, patients with ZIKV-CON, or the healthy control group (1-way ANOVA, F(2,106) = 0.7417, *p* = 0.4788) (eFigure 4G, links.lww.com/NXI/A760). IgG antibody reactivity was observed against different topographic domains within the cocultures. Sera from 4 of 52 (7.7%) patients with ZIKV-GBS contained IgG antibodies that bound the abaxonal membrane of a subset of myelinating SCs (Table 2), while showing no apparent reactivity against nonmyelinating SCs or axons (eFigure 5A and 5B, links.lww.com/NXI/A760).

**Table 2:**
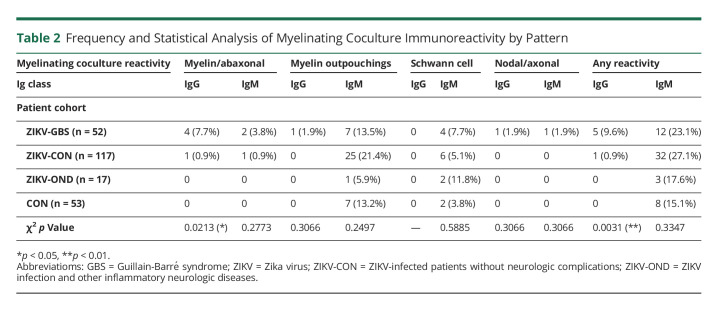
Frequency and Statistical Analysis of Myelinating Coculture Immunoreactivity by Pattern

IgG antibodies from the serum of 1 patient with ZIKV-GBS (male, 16–20 years old) were deposited on noncompact myelin at paranode-like regions of the node of Ranvier and Schmidt-Lanterman incisures (eFigure 5C, links.lww.com/NXI/A760). IgG antibodies from the serum of another ZIKA-GBS patient (patient A, male, 41–45 years old) labeled the axons of cultured neurons and was particularly concentrated at the nodal axolemma (eFigure 5D, links.lww.com/NXI/A760). These patterns of IgG antibody reactivity were never seen with the control sera. The serum of 1 patient with ZIKV-CON (patient B, female, 41–45 years old) also contained IgG antibodies, which gave an intense pattern of immunofluorescence over the abaxonal membranes of all myelinating SCs, and at the nodal microvilli (eFigure 5E, links.lww.com/NXI/A760), but no IgG antibody labeling occurred against sensory neuron or SC monocultures (data not shown). Overall, IgG antibody reactivity against myelinating cocultures was significantly more common in the sera of patients with ZIKV-GBS (5/52; 9.6%) compared all controls (1/187; 0.5%) (*p* = 0.0021, Fisher exact test) (see Table 2 for χ^2^ tests on multiple comparisons).

### Nerve Section Immunoreactivity

We also analyzed the reactivity of ZIKV-GBS (n = 30) and ZIKV-CON (n = 40) samples from the Barranquilla and Cucuta cohorts against macaque peripheral nerve. Overall, IgG and IgM reactivity against nerve tissue was frequently detected in patients with ZIKV-GBS (40%) and patients with ZIKV-CON (25%), although not statistically significant. We did, however, observe that 8 (26.7%) patients with ZIKV-GBS showed strong reactivity against nonmyelinating SCs (4 patients with IgG, 2 patients with IgM, and 2 patients with both IgG and IgM antibodies), whereas only 1 (2.5%) patient with ZIKV-CON showed immunoreaction of IgG (IgG, *p* = 0.0371; IgM, *p* = 0.0299) (data not shown).

### Combined Screening Results

Overall, 125 samples were tested against both isolated primary cells and myelinating cocultures. Only, 1/13 (16.0%) IgG-positive sera (*ZN005A*) and 8/50 (16.0%) IgM-positive sera were identified in both systems. The kappa statistics for inter-test agreement were 0.129 (IgG) and 0.084 (IgM).

### Assessment of Antibody-Mediated Axonopathy and Demyelination

We next investigated the pathologic potential of IgG autoantibodies in the 2 serum samples that reacted strongly against the cell cocultures: patient A (eFigure 5D, links.lww.com/NXI/A760) and patient B (eFigure 5E, links.lww.com/NXI/A760). The axonal and nodal binding of IgG antibodies present in patient A's serum was exclusively of the IgG1 subclass (data not shown). The addition of patient A's serum at a 1:100 dilution did not result in any axonal degeneration or other visible morphologic nerve injury in the presence of complement in 20% NHS compared with that caused by a known complement-fixing anti-ganglioside IgM-positive control antibody (data not shown). Antibodies reactive to myelinating SCs in patient B's serum were also predominantly of the IgG1 antibody subclass, with some IgG4 antibody subclass reaction against either SC processes or axons (data not shown).

**Figure 2:**
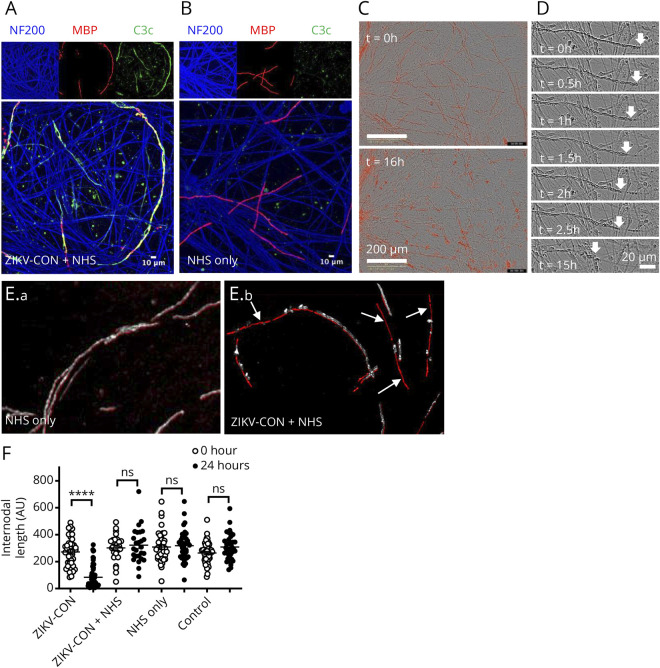
Complement-Dependent Demyelinating Activity of ZIKV Patient Sera The autoantibodies in the sera of ZIKV-CON patient B (*BQ008*) reacted with myelin internodes and fixed complement (C3c deposition; green) over myelin internodes (MBP; red) when complement was present from 20% normal human sera (NHS) (A) but not when only NHS was added to the cells (B). Counterstaining for NF200 (blue, axons) and MBP (red) is also shown. (C) Fluoromyelin labeling (red) from live imaging of a myelinated coculture before (above) and 16 hours after (below) incubation with patient B's serum at a 1:50 dilution in the presence of complement in 20% NHS, while (D) shows the results of time-lapse stills of the receding myelin internode junctions shown from right to left over time (arrows). (E) shows the overlaid example images of live myelin staining at baseline (red) and after 24 hours of incubation (silver) with patient B's serum with or without 20% NHS containing complement. (E.b) shows numerous myelin internodes are either lost completely or extensively fragmented after 24 hours (shown by arrows), while (E.a) without NHS containing complement the distribution of the myelin was essentially unchanged over 24 hours. (F) The mean internodal length was significantly reduced at 24 hours compared with baseline in the ZIKV-CON + NHS group only (****p < 000.1, 2-way ANOVA with Sidak correction); ns = not significant. MBP = myelin basic protein; ZIKV = Zika virus; ZIKV-CON = ZIKV-infected patients without neurologic complications.

The addition of patient B's sera at a 1:100 dilution to cocultures in the presence of 20% NHS resulted in serum complement deposition (C3c) around myelin internodes and myelin fragmentation without apparent axonal degeneration (Figure 2, A and B). Time-lapse imaging of fluoromyelin-stained internodes revealed retraction of the myelin from the node within hours of exposure to the anti-ZIKV serum in the presence of complement (Figure 2, C and D). After 24 hours, multiple myelin internodes were either lost completely or significantly fragmented compared with baseline (0 hour) (Figure 2E). ZIKV-CON serum treatment of the myelinating cocultures in the presence of complement in the 20% NHS, but not ZIKV-CON serum or NHS alone, led to a significant loss of myelin coverage and shortening of internode length (Figure 2F), thereby confirming the effect seen using the time-lapse imaging (Figure 2D). In a confirmatory experiment (eFigure 6A, links.lww.com/NXI/A760), the addition of myelin-binding IgG from patient B serum *BQ008* (1:50) to the cocultures for 24 hours also resulted in smaller myelin fragments (F[3,214] = 6.871, *p* = 0.0002, 2-way ANOVA; interaction between serum sample and complement with the Sidak multiple comparison test; *p* = 0.0002) (eFigure 6B, links.lww.com/NXI/A760) and a greater proportion of fragmented internodes (*p* < 0.0001, Fisher exact test) after the addition of 20% NHS (eFigure 6C, links.lww.com/NXI/A760), which was not observed with DRG/SC reactive ZIKV-GBS sample *BQ004* (M, 41–45 years) (eFigure 6A, links.lww.com/NXI/A760) and 2 other nonreactive ZIKV-CON samples *BQ010* (M, 36–40 years) and *BQ024* (F, 46–50 years). Neuronal density was not affected (eFigure 6D, links.lww.com/NXI/A760).

### Antigen Identification in Myelinating Cocultures

The nodal/axonal binding ZIKV-GBS sera of patient A (eFigure 5D, links.lww.com/NXI/A760) showed a similar binding pattern to sera from a separate study containing anti-CNTN1 antibodies.^28^ Both sera maintained a similar IgG antibody binding pattern when reacted with neuronal monocultures, thereby confirming the neuronal origin of both the unknown ZIKV-GBS autoantigen and CNTN1. By contrast, no antibody labeling was observed with healthy control serum (data not shown). In accordance with the nodal IgG antibody labeling observed in the myelinating cell cocultures, we tested for the nodal/paranodal candidate neurofascin (NF) in cell-based assays. Heterologous expression of NF155 and NF186 isoforms in an HEK cell-based assay confirmed their antigen-specific targeting by IgG1 antibodies in this ZIKV-GBS patient's sera (data not shown) who subsequently died due to their illness. This anti–pan-NFASC protein isoform IgG antibody autoreactivity pattern was not detected in any of the other ZIKV-GBS patients' or control sera.

### Immunoprecipitation and Mass Spectrometry (IP-MS)

We used a proteomic approach to identify candidate antigens from our live cell culture screening by isolating antigen-bound IgG antibodies from serum-treated cultures using protein G bead IP followed by high-resolution C18 reversed-phase chromatography and then MS analyses. We first validated this approach using patient A's serum in neuronal cell monocultures. Three potential antigens were identified as enriched in the IP: NFASC, NCAM1, and NCAM2, confirming that NFASC was a target antigen of patient A's serum.

**Figure 3:**
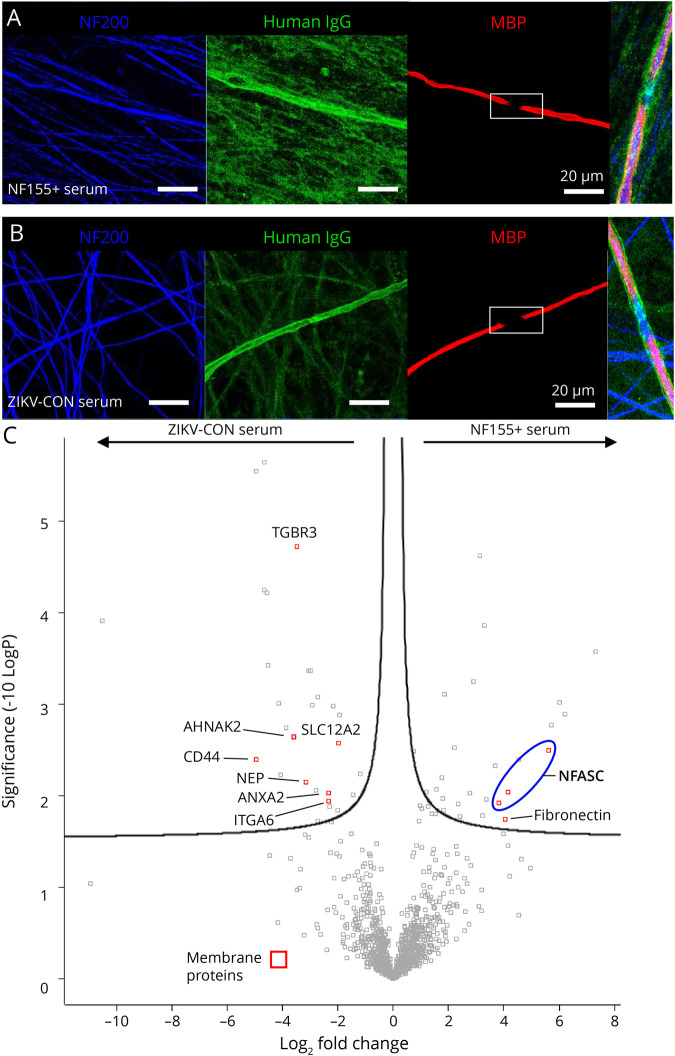
Identification of a ZIKV Patient's Autoantibodies Against Myelin Antigen: Immunoprecipitation and Peptide Mass Spectrometry Immunofluorescent photomicrographs of (A) a positive control patient's serum with autoantibodies against the known nerve antigen NF155 and (B) ZIKV-CON patient B's serum autoantibody reactions against unknown antigens enriched in abaxonal myelin, with higher magnifications of the nodal regions shown as insets (far right). Scale bars, 20 μm. Counterstaining for NF200 (blue) and MBP (red) is also shown. (C) Proteins identified in immunoprecipitates from ZIKV-CON patient B serum and NF155+ serum-treated coculture lysates. The *t* test of enriched proteins presented as a volcano plot. Significantly enriched membrane proteins are shown as red squares. NFASC protein isoforms from a human and rat database are grouped together (blue circle). AHNAK2 = desmoyokin; CD44 = P-glycoprotein 1; ITGA6 = integrin alpha 6. MBP = myelin basic protein; NEP = neprilysin; SLC12A2 = solute carrier 12/Na-K-2Cl cotransporter A2; TGBR3 = transforming growth factor receptor 3; ZIKV = Zika virus; ZIKV-CON = ZIKV-infected patients without neurologic complications.

The abaxonal SC binding by ZIKV-CON patient B's serum was compared with a patient with anti-NF155 antibodies by IP-MS, which showed a similar pattern of IgG autoantibody binding (Figure 3, A–C). A total of 28 proteins were significantly enriched by patient B's sera including immunoglobulin complement, and intracellular proteins, and 7 membrane-related proteins: ANXA2, TGFBR3, NKCC1, NEP, CD44, ITGA6, and AHNAK2 (Figure 3C).

Sera from 2 of the patients with ZIKV-GBS (patient C and patient D), which showed moderate or strong antibody reactivity against primary DRG neurons, and 1 from a healthy control were also taken forward for IP-MS analysis. Antigen-bound IgG antibodies from patient C's sera and patient D's sera or secondary IgG anti-IgM antibodies bound to patient D's IgM anti-neuronal autoantibodies were isolated from DRG neuron lysates by IP and processed for MS. In these experiments, 6 potential autoantigens (ALCAM, DPYSL2, NCAM1, CNTN1, L1CAM, and VINC) were identified as enriched by these anti-ZIKV sera compared with the control sera.

### Evaluation of Candidate Antigens

Candidate antigens identified by IP-MS were evaluated by either ELISA or heterologous expression in live cell-based assays. ELISAs using human recombinant prosaposin and GM3 (alone or in combination), sulfatide, multiple human laminin isoforms, and nidogen-1 failed to confirm these as targets of patient B's serum IgG autoantibodies.

Neither heterologous expression in HEK cells nor ELISAs revealed binding of ZIKV-CON patient B's serum IgG antibodies to any of the large number of other candidate proteins (see eMethods, links.lww.com/NXI/A760, for the list of plasmids). Furthermore, neither the IgG or IgM antibodies from ZIKV-GBS patient C or D reacted against any of the candidate antigens by heterologous expression in these cell-based assays. We additionally analyzed IgG and IgM autoantibody reactivity of anti-ZIKV sera against GAS6 and AXL proteins, which have been described as the receptor ZIKV virus entry to human neural cells and NrCAM, which shares 6 common peptide sequences with the ZIKV polyprotein. However, none of these sera reacted against these 3 candidate antigens or members of the AXL-GAS6 complex.

To account for the potential loss of antigen during the IP process, we subjected the lysates of myelinating cell cocultures to electrophoresis on an acrylamide gel, and after electroblotting onto nitrocellulose membranes, the reactions of patient B's serum autoantibodies were tested against these nerve cell antigens. Two bands of relative molecular weights of approximately 62 and 50 kDa were strongly detected by the serum IgG antibodies in the myelinated cell cocultured lysate, but not in the neuronal or Swann cell lysates (data not shown). Manual filtering for membrane proteins associated with SCs or myelination identified 3 potential target antigens: PRX, ITGA7, and GFRA1. However, no IgG antibody reactivity to these particular antigens was observed using patient B's serum on transiently transfected cell-based assays.

### Chemical and Enzymatic Characterization of Target Antigens

**Figure 4:**
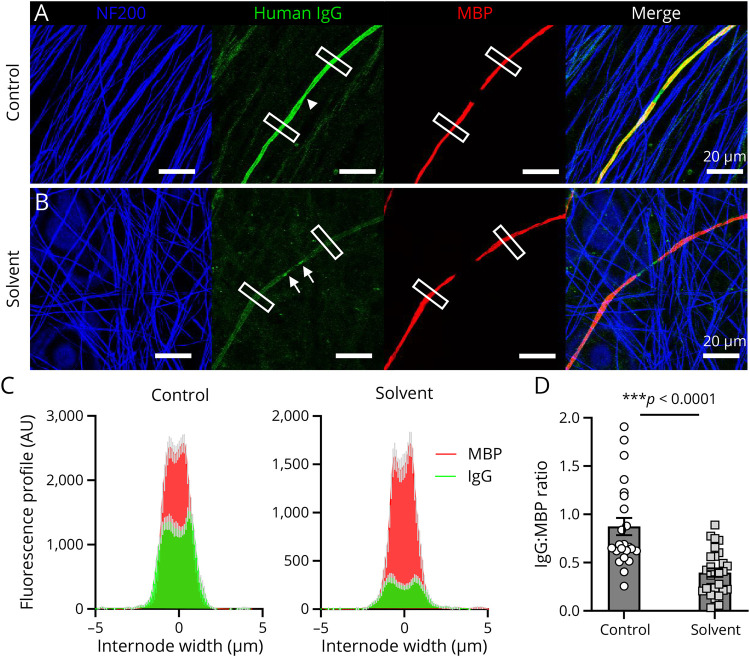
Removal of ZIKV-CON Patient B's Serum IgG Autoantibody Target Antigen(s) in Myelinated Sensory Neuron Cultures by Lipid Extraction (A) Immunofluorescent photomicrographs of ZIKV-CON patient B's serum IgG autoantibodies showing strong staining with untreated (control) abaxonal membranes of all myelinating SCs, which was particularly intense at the Schwann cell microvilli overlying the node of Ranvier (arrowhead) while (B) weakly staining these same cells after solvent treatment (solvent). The control reactions for neuronal (NF200) and myelin (MBP) markers remained unchanged. For this study, the serum-treated fixed cultures were incubated with a mixture of chloroform, methanol, and water at a ratio of 4:8:3 for 1 hour on ice to remove glycolipids. The white boxes indicate regions of fluorescence profiling per internode (5 × 20 μm). Arrows indicate weak paranode-like IgG antibody binding after lipid extraction. (C) Fluorescence intensity profiles of the human IgG autoantibodies and MBP immunolabeling across the internodes of control and solvent-treated cultures. Data are presented as mean (± SEM in gray) of 2 internodes per field of view, 12–14 fields of view from 2 independent experiments. (D) Quantification of IgG:MBP fluorescence ratio. Student unpaired *t* test, *p* < 0.0001, *t* = 5.053, n = 24–28 internode profiles per group. MBP = myelin basic protein; ZIKV = Zika virus; ZIKV-CON = ZIKV-infected patients without neurologic complications.

The intense labeling of the patient B's serum autoantibodies in the lipid-rich membrane and nodal microvilli of myelinating SCs (Figure 4A) led us to further investigate the target antigen characteristics using a biochemical approach. Solvent extraction of lipids from serum-treated and fixed myelinated cocultures with a mixture of chloroform, methanol, and water as described previously^29^ significantly reduced the amount of patient B's serum IgG antibodies, which bound to the abaxonal SC membranes (Figure 4, B–D) (*p* < 0.0001, Student *t* test), whereas the reactivity of commercial antibodies directed against NF200 and MBP antigens was retained (Figure 4, B and C). Solvent extraction did not affect labeling of human IgG autoantibodies to the GPI-linked protein CNTN1 (*p* = 0.256, *t* = 1.223, df = 8, Student unpaired *t* test, n = 5 coverslips per group; data not shown). Limited IgG antibody labeling remained at the paranodes (Figure 4B). Binding of patient B's IgG antibodies was not, however, affected by prior neuraminidase treatment of the cell cocultures (*p* = 0.3605, *t* = 0.9340, n = 12 internode profiles per group, Student unpaired *t* test) compared with anti-GD2, anti-GD3 anti-disialosyl ganglioside antibody–positive controls (*p* < 0.001 and *p* < 0.0001, unpaired Student *t* test; data not shown), suggesting that the target antigen was not a sialylated glycolipid.

## Discussion

Our study found that the humoral immune response in ZIKV-GBS cases was diverse and heterogeneous. We found neither a common antigen nor a common pattern of serum immunoreactivity against peripheral nerve structures despite all these patients with ZIKV-GBS sharing a common prodromal infection.

Consistent with a recent study of a modest cohort of patients with ZIKV-associated GBS from Brazil,^11^ we observed that a small number of patients with GBS had elevated anti-glycolipid IgG antibodies. However, we did not find a significant statistical association of ZIKV-GBS patient cases with any anti-glycolipid antibody signature, thereby supporting the results from a recent cohort study from Northeast Brazil.^30^ In this study, we did not perform any additional screening for *Campylobacter*. However, in the Cucuta cohort, IgG antibodies for *Campylobacter jejuni* were measured and found to be present in 24.14% of patients with GBS and in 16.22% of controls, with no significant difference.^21^ Thus, in accordance with our glycoarray data, a humoral response to *C jejuni* is possible in a minority of patients but unlikely to be a key driver of neurologic disease in ZIKV-GBS.

Analysis of the ZIKV polyprotein showed that it contained peptide sequences that were also found in human proteins and that are known to be targets of inflammatory neuropathy autoantibodies.^31^ However, although all ZIKV patient samples tested contained anti-ZIKV IgG antibodies, anti-nerve reactive autoantibodies showed a more discrete distribution, suggesting that any nerve-related auto-antigenicity was probably not a consequence of humoral immunity to ZIKV antigens per se. Despite the high level of antigenic similarity between flaviviruses, we did not observe any selective nerve cell reactive autoantibodies in the DENV-infected control (DENV-CON) sera, in keeping with the rarity of GBS associated with recent DENV infections.^32^ Although we observed 1 case of anti–pan-NFASC protein isotype IgG1 autoantibodies in our Colombian cohort (ZIKV-GBS patient A), prodromal infection is not a consistent feature of this rare and potentially fatal peripheral neuropathy subtype.^33^

By using a comprehensive series of peripheral nerve cell culture models and antigen expression systems, we reveal a surprisingly heterogeneous autoantibody response with potential nerve reactivity in patients' sera. Overall, IgG reactivity to rat SCs and the myelinating cocultures was significantly higher, albeit infrequent, in the ZIKV-GBS patient group compared with all controls. IgM antibody reactivity against rat DRG neurons and rat SCs was also significantly higher in patients with ZIKV-GBS than in the control group. Moreover, we also observed a higher proportion of ZIKV-GBS sera reacting against nonmyelinating SCs in peripheral nerve sections, compared with the ZIKV-CON group. On the other hand, IgM antibody immunoreactivity to myelin outpouchings, a feature reminiscent of the myelin foldings, swellings, or tomaculae sometimes seen in certain hereditary neuropathies,^34^ was remarkably common in both the ZIKV-GBS patient and control groups. The significance of these structures in the coculture system is unclear.

Over 50% of our ZIKV-GBS cohorts received IVIg treatment at some point before sampling. We therefore asked whether artificially elevated serum IgG levels may lead to an increase in false positives on the cell-based assays. Although overall IgG levels were on average slightly higher in the ZIKV-GBS group, IgG levels in the ZIKV-CON and ZIKV-GBS groups fell within the same range (1.3–7.9 mg/mL), with the exception of a single outlier (Cali-021) that did not receive a positive score on the coculture screen, and there was no correlation between overall IgG levels and cell culture positivity. Although this finding supports the selectivity of detection of nerve reactive positivity in the live cell culture screen despite IVIg treatment, we cannot exclude a loss in sensitivity of detection leading to false negatives in post-IVIg samples with originally low level reactivity.^35^

Only 1 patient's serum sample was identified as strongly immunoreactive in both myelinating cocultures and DRG/SC assays. The low concordance suggests key antigenic differences between the acute primary culture and long-term myelinating culture assays. Efforts to reduce the background immunofluorescence signal, as well as further dilution series of serum samples, may improve the fidelity of the live culture systems to parse GBS from non-GBS nerve immunoreactivity.

One patient (ZIKV-CON patient B), with an uncomplicated ZIKV infection, displayed striking IgG antibody reactivity against the abaxonal membrane of myelinating SCs that was particularly enriched at the nodal villi. These IgG1 subclass autoantibodies induced profound demyelination in our in vitro model system via a complement-dependent pathway, despite the patient lacking neurologic symptoms. This apparent functional discrepancy might be accounted for by a number of factors: the use of rodent SCs in the coculture system and a potential lack of cross-reactivity to human SCs; by the target antigen(s) remaining hidden from circulating antibodies in situ; or by a functional loss of complement signaling in this patient, although the latter is rare and usually accompanied by a spectrum of autoimmune presentations,^36^ which were lacking in this case.

By using IP-MS in sera-treated myelinating cocultures and DRG neurons, we revealed a number of candidate antigens related to SC membranes, myelin formation, or neural cell adhesion. However, we could not confirm any candidate antigen reactivity by these patients' IgG or IgM antibodies by either the ELISA or cell-based assays, reinforcing the challenges of antigen identification in the inflammatory neuropathies.^37^ Nevertheless, the selective patterns of immunoreactivity, such as those against nodal structures, axons, or myelin internodes in the myelinating cocultures, were not observed in any control samples. This suggests that the near-native antigen conformation in the cell coculture system is a high-fidelity substrate for screening potentially pathogenic patients' serum IgG autoantibodies.^17-19^

A limitation of our study is that our antigen identification protocols were optimized for proteins and not for lipids. In fact, solvent-based lipid extraction significantly reduced reactivity of patient B's IgG autoantibody reactions against myelinating SCs in the coculture system, suggesting that a lipidomic approach may be required. It is also possible that certain protein antigens were not detectable, either due to poor IP or lack of representation in the peptide database used to compile our list of candidate antigens. An alternative approach is offered by high-throughput antibody screening techniques such as the newly described Rapid Extracellular Antigen Profiling technique,^38,39^ or chip-based proteome profiling,^40^ which has recently offered insights into the autoantibody repertoire of patients with chronic inflammatory demyelinating polyradiculoneuropathy.^41^

Our findings mirror our recent screening of a separate cohort of 100 patients with GBS, where we report a similarly heterogeneous reactivity of their IgG and IgM antibodies to nerve-related antigens.^20^ The narrow definition of the present GBS cohort, in which all patients associated with the same infectious insult, suggests that the heterogeneity of the humoral immune responses in GBS may be a core feature of the disease and not necessarily due to the heterogeneity of the patient cohorts or prodromal infections.

## Glossary

ALCAM: activated leukocyte cell adhesion molecule
ANOVA: analysis of variance
ANXA2: annexin A2
AMAN: acute motor axonal form of GBS
AXL: Axl receptor tyrosine kinase
CNTN1: contactin 1
DENV: dengue virus
DPYSL2: dihydropyrimidinase-related protein 2
DRG: dorsal root ganglia
Fc: fragment crystalizable region (of antibody)
GBS: Guillain-Barré syndrome
GFRA1: glial cell-derived neurotrophic factor family receptor alpha-1
hiPSC: human induced pluripotent stem cell
HRP: horse radish peroxidase
IgG: immunoglobulin G
IgM: immunoglobulin M
ITGA7: integrin alpha 7
L1CAM: neural cell adhesion molecule L1
MS/MS: tandem mass spectrometry
MOPS: 3-(N-morpholino)propanesulfonic acid
NCAM1: neural cell adhesion molecule 1
NEP: neprilysin
NF155: neurofascin 155 isoform
NF186: neurofascin 186 isoform
NF200: neurofilament heavy chain (200 kDa)
NFASC: neurofascin
NHS: normal human serum
NKCC1: solute carrier Na-K-2Cl cotransporter 1
NrCAM: neuronal cell adhesion molecule
PRX: periaxin
SC: Schwann cell
SEM: standard error of the mean
TGFBR3: transforming growth factor receptor type 3
VINC: vinculin
ZIKV: Zika virus
ZIKV-CON: ZIKV-infected patients without neurologic complications
ZIKV-OND: ZIKV infection and other inflammatory neurologic diseases
